# The EPH/Ephrin System in Gynecological Cancers: Focusing on the Roots of Carcinogenesis for Better Patient Management

**DOI:** 10.3390/ijms23063249

**Published:** 2022-03-17

**Authors:** Iason Psilopatis, Alexandros Pergaris, Kleio Vrettou, Gerasimos Tsourouflis, Stamatios Theocharis

**Affiliations:** 1 First Department of Pathology, Medical School, National and Kapodistrian University of Athens, 75 Mikras Asias Street, Bld 10, Goudi, 11527 Athens, Greece; iason.psilopatis@charite.de (I.P.); alexperg@yahoo.com (A.P.); kliovr1@gmail.com (K.V.); gtsourouflis@med.uoa.gr (G.T.); 2 Charité-University School of Medicine, Augustenburger Pl. 1, 13353 Berlin, Germany

**Keywords:** EPHs, ephrins, ovarian cancer, endometrial cancer, cervical cancer

## Abstract

Gynecological cancers represent some of the most common types of malignancy worldwide. Erythropoietin-producing hepatocellular receptors (EPHs) comprise the largest subfamily of receptor tyrosine kinases, binding membrane-bound proteins called ephrins. EPHs/ephrins exhibit widespread expression in different cell types, playing an important role in carcinogenesis. The aim of the current review was to examine the dysregulation of the EPH/ephrin system in gynecological cancer, clarifying its role in ovarian, endometrial, and cervical carcinogenesis. In order to identify relevant studies, a literature review was conducted using the MEDLINE and LIVIVO databases. The search terms *ephrin*, *ephrin receptor*, *ovarian cancer*, *endometrial cancer*, and *cervical cancer* were employed and we were able to identify 57 studies focused on gynecological cancer and published between 2001 and 2021. All researched ephrins seemed to be upregulated in gynecological cancer, whereas EPHs showed either significant overexpression or extensive loss of expression in gynecological tumors, depending on the particular receptor. EPHA2, the most extensively studied EPH in ovarian cancer, exhibited overexpression both in ovarian carcinoma cell lines and patient tissue samples, while EPHB4 was found to be upregulated in endometrial cancer in a series of studies. EPHs/ephrins were shown to exert their role in different stages of gynecological cancer and to influence various clinicopathological parameters. The analysis of patients’ gynecological cancer tissue samples, most importantly, revealed the significant role of the EPH/ephrin system in the development and progression of gynecological cancer, as well as overall patient survival. In conclusion, the EPH/ephrin system represents a large family of biomolecules with promising applications in the fields of diagnosis, prognosis, disease monitoring, and treatment of gynecological cancer, with an established important clinical impact.

## 1. Introduction

**Figure 1:**
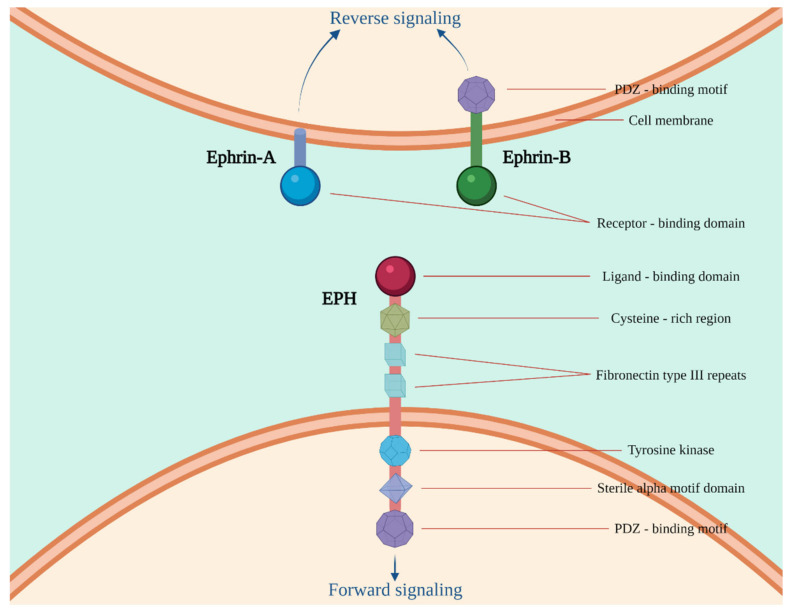
Structure of the erythropoietin-producing hepatocellular receptor (EPH)/ephrin molecules. Ephrin-A ligands are anchored to the plasma membrane by a glycosylphosphatidylinositol anchor, whereas ephrin-B ligands contain a transmembrane domain and a short cytoplasmatic tail. EPHs/ephrins, upon activation, exert their physiological as well as their tumor-promoting and tumor-suppressive functions through complex molecular pathways inside the cytoplasm. Forward signaling is conducted through EPH’s interaction with a number of different biomolecules and pathways, such as GTPases of the Rho and Ras family, focal adhesion kinase (FAK), and the pathways of the Janus kinase (JAK)-signal transducer and activator of transcription (STAT), as well as the phosphoinositide 3-kinase (PI3K). In backward signaling, upon the phosphorylation of ephrins, different proteins that contain Src Homology 2 (SH2) or PDZ domain, such as Grb4, interact with the ephrin and transmit the signal downstream [14]. Created with BioRender.com.

Erythropoietin-producing hepatocellular receptors (EPHs) constitute the largest subfamily of receptor tyrosine kinases (RTK) that bind membrane-bound proteins called ephrins [1]. EPHs are categorized into two subfamilies, EPHAs and EPHBs, based on their structural homology and preferential binding affinities to their ephrin-A and ephrin-B ligands, respectively [2,3]. In humans, nine EPHA receptors (EPHA1-8, 10) that bind five ephrin-A ligands (ephrin-A1–5), along with five EPHB receptors (EPHB1–4, 6) that interact with three ephrin-B ligands (ephrin-B1–3), have been described (Table 1) [4,5,6]. Ephrin-A ligands generally interact with EPHAs via a glycosylphosphatidylinositol anchor on plasma membranes, whereas EPHBs are bound by ephrin-B ligands and tethered to the membrane by a transmembrane domain [7]. EPH–ephrin interaction triggers a response in the cytoplasm of the receptor-expressing cell (called forward signaling) as well as a molecular cascade in the cytoplasm of the ephrin-bearing one, termed backward signaling (Figure 1). The complexity of the EPH/ephrin signaling system is further underlined by the established ephrin-independent EPH activation by a number of molecules, such as Ephexin1, Ephexin4 [8], and progranulin [9], and even by EPH–EPH interaction [10]. EPHs and their ligands are widely expressed in different cell types and participate in various physiological functions, most of which are directly involved in tumorigenesis and cancer progression [11,12].

**Table 1:** Molecular function of EPH/ephrin genes and proteins in carcinogenesis [13].

EPHs/Ephrins	Molecular Function in Carcinogenesis
**EPHA1**	High affinity to ephrin-A1. Cell attachment induction to the extracellular matrix. Cell spreading and motility inhibition through integrin-linked protein kinase (ILK) regulation and Ras Homolog Family Member A (RHOA) and Rac Family Small GTPase (RAC) downstream.
**EPHA2**	Activation by the ligand ephrin-A1. Regulation of cell migration, integrin-mediated adhesion, proliferation and differentiation through Desmoglein-1, and inhibition of the extracellular signal-regulated kinases 1/2 (ERK1/2) signaling pathway.
**EPHA3**	High affinity to ephrin-A5. Regulation of cell–cell adhesion, cytoskeletal organization and cell migration.
**EPHA4**	Activation by ephrin-A1 and -B3. Cell morphology modulation and integrin-dependent cell adhesion through regulation of the RAC, Ras-related protein (RAP) and Rhodopsin (Rho) GTPases activity.
**EPHA5**	High affinity to ephrin-A5.
**EPHA6**	Contact-dependent bidirectional signaling into neighboring cells.
**EPHA7**	High affinity to ephrin-A5.
**EPHA8**	Activation by ephrin-A2, -3, and -5.
**EPHA10**	Activation by ephrin-A3, -4, and -5.
**EPHB1**	Activation by ephrin-B1, -2, and -3. Cell migration regulation through activation of the ERK signaling pathway. Cell adhesion regulation through activation of the c-Jun N-terminal kinase (JNK) signaling cascade.
**EPHB2**	Activation by ephrin-B2.
**EPHB3**	Activation by ephrin-B2.
**EPHB4**	Activation by ephrin-B2. Regulation of cell adhesion and migration. Cellular repulsion and segregation control.
**EPHB6**	High affinity to ephrin-B1, and -2. Cell adhesion and migration modulation, inhibition of JNK activation, T-cell receptor-induced IL-2 secretion, and CD25 expression upon stimulation with ephrin-B2.
**ephrin-A1**	Induction of RAC1 GTPase activation and vascular endothelial cell migration and assembly.
**ephrin-A2**	Contact-dependent bidirectional signaling into neighboring cells.
**ephrin-A3**	Contact-dependent bidirectional signaling into neighboring cells.
**ephrin-A4**	Contact-dependent bidirectional signaling into neighboring cells.
**ephrin-A5**	Compartmentalized signaling induction within a caveolae-like membrane microdomain when bound to the extracellular domain of its cognate receptor through the activity of the Fyn tyrosine kinase. Cell-cell adhesion and cytoskeletal organization regulation.
**ephrin-B1**	Contact-dependent bidirectional signaling into neighboring cells.
**ephrin-B2**	Cellular repulsion and segregation control.
**ephrin-B3**	Contact-dependent bidirectional signaling into neighboring cells.

Pergaris et al., recently reviewed the clinical impact of the EPH/ephrin family’s expression in solid tumors, reporting their key role in the development and progression of head and neck, thoracic, skin, gastrointestinal tract, urinary tract, gynecological, and pediatric cancer [15]. Ovarian, endometrial, and cervical tumors represent the main types of gynecological cancer (GC). More than 90,000 women were diagnosed with GC each year between 2012 and 2016 in the USA, with the incidence rate varying by cancer type and race [16]. In this review, we investigate the dysregulation of the EPH/ephrin system in gynecological tract neoplasia, incorporating research works that explored their role in carcinogenesis via both in vitro and in vivo studies. The literature review was conducted using the MEDLINE and LIVIVO databases. The search terms *ephrin*, *ephrin receptor*, *ovarian cancer*, *endometrial cancer*, and *cervical cancer* were employed and we were able to identify 57 studies focused on gynecological cancer and published between 2001 and 2021.

## 2. Ovarian Cancer

Ovarian cancer (OC) is the leading cause of death from gynecological malignancies and the fifth most common cause of tumor-related deaths in women in the United States. For 2021, the American Cancer Society estimates the incidence of OC at 21,410 cases and OC-related deaths at 13,770 in the USA [17]. OC includes several histological types. Epithelial carcinomas comprise the vast majority of OCs, with high-grade serous OC representing the most common morphological subtype [18]. Lack of specific symptoms of the disease at its early stages is a significant factor contributing to the typical advanced stage of the tumor at diagnosis, after metastasis has already occurred [19]. Consequently, the five-year survival rate for women diagnosed with invasive epithelial OC in a distant Surveillance, Epidemiology, and End Results (SEER) stage is 31% [20]. In spite of substantial research efforts, the molecular mechanisms of OC’s origin, initiation, and progression still remain largely unclear [21]. Given the lack of effective diagnostic tools and treatment strategies, it is hence of utmost importance to identify new molecular markers involved in the pathogenesis of OC, with a view to offering novel, targeted, biological therapeutic approaches.

A number of EPH/ephrin system members have been shown to play an important role in OC, including EPHA1, EPHA2, EPHA4, EPHA5, EPHA8, EPHB1, EPHB2, EPHB3, EPHB4, EPHB6, ephrin-A1, ephrin-A3, ephrin-A5, ephrin-B1, and ephrin-B2.

**Figure 2:**
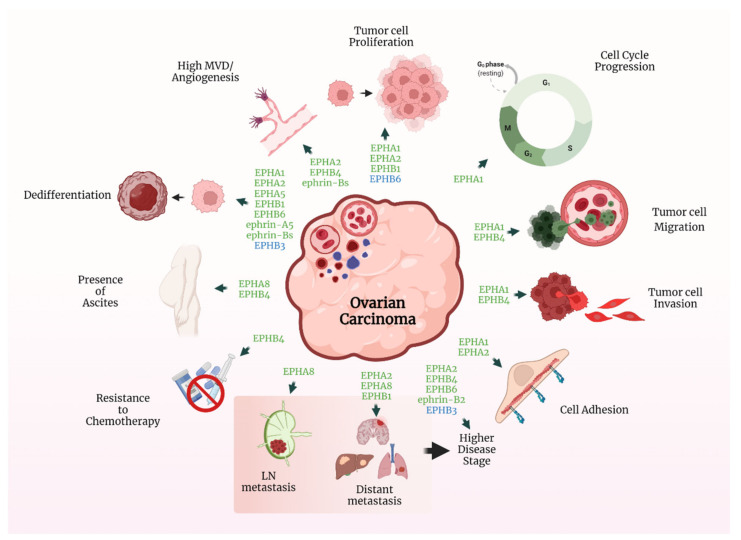
Key roles of the EPH/ephrin system in OC pathogenesis. Green font: EPHs/ephrins that promote each described process. Blue font: EPHs/ephrins that inhibit the specific action. Created with BioRender.com.

Figure 2 illustrates some of the key roles of the EPH/ephrin system in OC pathogenesis.

### 2.1. The EPH/Ephrin System in OC Cell Lines and Human Xenograft Models

Many studies have investigated the role of the EPH/ephrin system in OC cell lines (Table S1) and human xenograft models. Cui et al., knocked down the *EPHA1* gene using the CRISPR/CAS9 technique. The inactivation of *EPHA1* suppressed many aggressive properties and resulted in G0/G1 cell cycle arrest, reduction of the cellular adhesion capacity, and inhibition of the migration capacity, proliferation, transwell invasion, and activity of the matrix metalloproteinase (MMP)-2 and c-MYC signaling pathways in SKOV3 and COV504 OC cells [22]. On the contrary, Jin et al., reported that EPHA1 expression was negative in HO8910 and weakly positive in A2780 OC cells, with the proliferation rate being significantly reduced in OC cells after transfection with EPHA1 plasmid compared with cells transfected with mock plasmid or untreated ones. An alteration in apoptosis could not, however, be detected in these groups [23].

EPHA2 has been described to promote the growth of OVK-18 cells [24]. Thaker et al., evaluated EPHA2 expression in OC cell lines by Western blot analysis. EG, 222, and SKOV3 OC cell lines overexpressed EPHA2, whereas A2780-PAR and HIO-180 exhibited low to absent EPHA2 expression [25]. Both OVCAR3 and SKOV3 cells demonstrated strong EPHA2 and ephrin-A1 mRNA expression, as detected through reverse transcription polymerase chain reaction (RT-PCR) and Western blot [26]. Moreover, EPHA2 overexpression promoted cell–extracellular matrix attachment in A2780 cells, increased anchorage-independent cell growth in vitro, promoted tumorigenesis in an orthotopic mouse model of OC, and resulted in enhanced microvessel density (MVD) [27].

EPHB2 demonstrated only small variations in RNA expression across OC cell lines. Promoter hypermethylation of EPHB2, EPHB3, and EPHB4 did not, however, seem to play an important role in ovarian tumors, as reported by Wu et al. [28]. Davidson et al., conducted affymetrix U133A array analysis for angiogenic gene expression in multiple OC cell lines. The genes encoding *ephrin-B2* and *EPHB4* were upregulated in mutant TP53 cells; *ephrin-B2* was upregulated in the A2780 line; and *EPHB4* was overexpressed in the larger pool of mutant TP53 lines. In contrast, EPHB2 showed high expression levels in wild-type TP53 cell lines, while overexpression of ephrin-A3 was induced by hypoxia [29]. EPHB4 was found to be highly expressed in OC cell lines and to regulate cell migration and invasion. Treatment of Hoc-7 cells with progesterone led to a dose-dependent reduction in EPHB4 expression, while inhibition of EPHB4 by specific siRNA or antisense oligonucleotides resulted in reduced viability and led to apoptosis as well as activation of the death receptor caspase pathway in this OC cell line. Interestingly, EPHB4 synthetic antisense oligodeoxynucleotides significantly inhibited tumor growth in mice-bearing human OC xenografts [30]. Ma et al., transfected antisense EPHB4 and shRNA vectors into A2780 and SKOV3 cells. Co-transfection with both vectors could inhibit growth, induce apoptosis, and reduce invasive ability of OC cells, accompanied by downregulation of EPHB4 and the PI3K/Akt/mTOR pathway [31].

Ephrin-A1 expression was shown to be induced in OC cells by the pleotropic transcription nuclear factor kappa B (NFκB) after stimulation with tumor necrosis factor alpha (TNF-α) and interleukin 1 beta (IL-1β) [32]. Ephrin-A3 expression was upregulated by hypoxia and promoted endothelial cell migration and adhesion in OC cell lines [29]. Unlike ephrin-A1, Jukonen et al., suggested that endogenous ephrin-A5 is an inefficient activator of EPHA2-pY588 signaling and receptor internalization in OC cells [33]. 

Ephrin-B2 was found to be overexpressed in OC cell lines [29].

Table 2 summarizes the role of the EPH/ephrin system in OC cell lines and human xenografts.

### 2.2. The EPH/Ephrin System in OC Patient Tissue Samples

The upregulation of EPHA1 in OC tissues reveals its implication in disease onset and progression [34]. EPHA1 expression has been reported to be increased by more than 10-fold in OC specimens through quantitative real-time (RT-PCR) [35] and to predispose OC patients to adverse clinical outcomes [36].

Similarly, EPHA2 has led to overexpression in OC tissues in a series of studies [24,25,26,35,37]. High levels of EPHA2 protein expression have been associated with a higher tumor grade, advanced disease stage, high MVD, strong stromal reaction and epithelial matrix metalloproteinase (MMP)-9, epithelial MMP-2, and epithelial membrane-type 1 MMP expression, as well as shorter patient survival [25,26,37]. Specifically, EPHA2 processing, activated by membrane-type 1 MMP, was involved in malignant transformation of ovarian tumors [38].

*EPHA4* expression in OC patients correlated with early relapse and showed an adverse clinical association when expressed in both OC tumor cells and tumor-associated macrophages [36].

On the contrary, loss of EPHA5 immunohistochemistry (IHC) expression in OC cases was shown to be associated with tumor grade, International Federation of Gynecology and Obstetrics (FIGO) stage, and poor patient outcome [39].

Liu et al., employed a tissue microarray IHC analysis and detected high EPHA8 protein expression in 44.80% of OC tissues, but only 6.67–15% of normal or benign ovarian tissues. A high EPHA8 protein level correlated with older age at diagnosis, higher FIGO stage, positive lymph nodes (LNs), the presence of metastasis, positive ascitic fluid, and higher serum CA-125 levels [40].

Loss of EPHB1 protein expression in OC tissue specimens was associated with higher tumor grade, the presence of metastasis, a high proliferative index (assessed by Ki67 expression), and significantly worse overall survival (OS) [41]. OC patients older than 60 years of age exhibited higher EPHB2 expression and poorer survival compared with younger ones [42]. On the other hand, Gao et al., performed IHC staining of EPHB3 and underlined that EPHB3 protein expression is significantly reduced in OC specimens, as well as negatively associated with histological grade and FIGO stage [43]. EPHB4 expression was shown to be upregulated in OC and correlated with an adverse clinical outcome, advanced disease stage, the presence of ascites, poorer OS, and poorer response to chemotherapy [30,36,42,44].

The expression of EPHB6 was associated with grade and TNM stage, and negatively correlated with Ki67 expression levels in OC samples, while patients with low EPHB6 protein expression exhibited a poorer outcome [45].

Elevated levels of ephrin-A1 expression led to a more aggressive tumor phenotype and were associated with poor survival in OC specimens [42]. High ephrin-A5 expression correlated with a more aggressive subtype of OC, as well as with poor patient OS [33,35,46].

High-grade OCs showed the greatest ephrin-B expression, with a strong correlation found between ephrin-B expression and MVD, disease recurrence, and a decrease in OS [47]. Schaner et al., investigated the genes that are more highly expressed in ovarian than breast carcinomas and identified *ephrin-B1* as one of the best discriminators more highly expressed in OC [48]. Ephrin-B2 was overexpressed in OC tissue samples, significantly increased with clinical stages, and correlated with poor survival [44]. Ephrin-B3 has been described to correlate with EPHB4 expression in ovarian tumor specimens [49].

Table 3 summarizes the role of the EPH/ephrin system in OC tissue samples.

## 3. Endometrial Cancer

With an estimate of approximately 66,570 newly diagnosed cases of cancer of the uterine body and about 12,940 deaths from cancers of the body of the uterus in 2021, endometrial cancer (EC) represents the most common malignant tumor of the female genital tract in the USA [50]. Depending on their development from atypical endometrial hyperplasia or atrophic endometrium, ECs can be divided into estrogen-dependent type I and type II nonendometrioid EC, respectively [51]. Even though the combination of surgery and chemotherapy as the first-line therapeutic regimen has showed high response rates, the duration of response does not last long, with a grim prognosis for patients with advanced EC and low five-year survival rates in the case of distant metastasis [52,53]. As such, a better understanding of the mechanisms controlling endometrial carcinogenesis is imperative in order to facilitate the development of novel prognostic and therapeutic tools and improve the clinical management of EC.

EPHs/ephrins that have been shown to play an important role in EC include EPHA2, EPHB3, EPHB4, ephrin-A1, and ephrin-B2.

**Figure 3:**
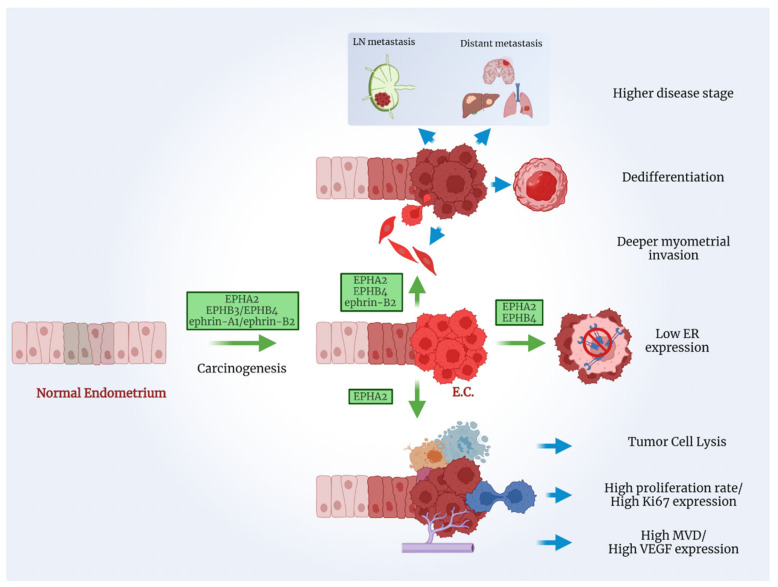
Members of the EPH/ephrin system promote various steps of EC tumorigenesis. Created with BioRender.com.

Figure 3 presents some of the oncogenic actions of the EPH/ephrin system in EC.

### 3.1. The EPH/Ephrin System in EC Cell Lines and Human Xenograft Models

EPHA2 protein expression was reported to be upregulated in EC cell lines [54]. EPHA2 has also been speculated to influence tumor cell lysis and contribute to susceptibility to Vδ1 γδ T cells’ cytotoxic reactivity [55].

EPHB3 and EPHB4 were shown to be overexpressed in EC cell lines through genome-wide microarray-based comparative genomic hybridization (aCGH) technology [54].

Ephrin-A1 induced intercellular dissociation, stimulated cell attachment, and inhibited cell aggregation through the EPHA receptor pathway in human EC-derived Ishikawa cells [56,57].

Table 4 summarizes the role of the EPH/ephrin system in EC cell lines (Table S1) and human xenografts.

### 3.2. The EPH/Ephrin System in EC Patient Tissue Samples

EPHA2 protein expression was reported to be upregulated in EC tissue samples, while its overexpression was significantly associated with high tumor stage and grade, increased depth of myometrial invasion, low estrogen receptor (ER) and progesterone receptor (PR) expression, high Ki67 index, high vascular endothelial growth factor (VEGF) expression, high MVD counts, and shorter disease-specific survival [58,59].

High EPHB4 expression positively correlated with histological tumor grade, adverse clinical disease stage, post-menopausal stage of the patient, dedifferentiation, myometrial invasion, ER expression, and poor survival rates in EC tissue samples [60,61,62,63,64].

Ephrin-B2 has been reported to be overexpressed in EC cells and to correlate with higher histological grade, adverse clinical disease stage, dedifferentiation, myometrial invasion, and poor survival rates [60,62,63,64]. Furthermore, ephrin-B2 activated EPHB4 receptor kinase through autocrine and/or paracrine activation [63].

Table 5 summarizes the role of the EPH/ephrin system in EC tissue samples.

## 4. Cervical Cancer

Cervical cancer (CC) used to be one of the most common causes of cancer death in female patients, but the death rate dropped significantly due to vaccination against HPV, as well as increased use of screening technology such as PAP and HPV tests. For 2021, the American Cancer Society estimates the CC incidence at 14,480 cases and CC-related deaths at 4290 [65]. Even though the tumorigenesis of CC is mostly associated with persistent infection with high-risk human papillomavirus (HPV) strains, growing evidence suggests that the pathophysiology of invasive CC involves dysregulation of a range of normal factors and cervical epithelium deterioration [66]. Treatment options vary by CC stage and range from radical hysterectomy to radiation with or without chemotherapy [67]. Nevertheless, the five-year survival rates for advanced CC patients are far from satisfactory, with the percentage of women diagnosed with invasive CC in a distant SEER stage reaching 17% [68]. Therefore, novel molecular markers that improve the clinical outcome could be of the highest value in developing individualized therapeutic approaches to CC.

EPHA2, EPHB2, EPHB4, ephrin-A1, and ephrin-B2 have been shown to play an important role in CC.

### 4.1. The EPH/Ephrin System in CC Cell Lines and Human Xenograft Models

EPHA2 protein expression exhibited upregulation in CC cell lines, leading to increased cell proliferation and migration of CC. EPHA2 knockdown increased the cellular susceptibility to epirubicin in CC cells and suppressed CC growth in a xenograft mouse model [69].

EPHB2 was also highly expressed in CC cell lines and promoted CC progression by inducing cell migration and invasion as well as the epithelial–mesenchymal transition (EMT) through R-Ras activation [70]. EPHB2 was identified as a direct target of microRNA-204 in CC cells [71].

Table 6 summarizes the role of the EPH/ephrin system in CC cell lines (Table S1) and human xenografts.

### 4.2. The EPH/Ephrin System in CC Patient Tissue Samples

EPHA2 protein expression exhibited upregulation in CC tissue samples, while its overexpression was associated with cyclin-dependent kinase 6 protein expression, invasion depth, LN metastasis, clinicopathological disease stage, and OS [69,72,73].

EPHB2 was highly expressed in CC tissue specimens and positively correlated with R-Ras expression [70,74].

Two large studies investigated the expression of EPHB4 through IHC and real-time RT-PCR in CC tissue samples and reported that high EPHB4 expression correlated with adverse clinical disease stage, larger tumor size, LN metastasis, high MVD, and poor patient OS [75,76].

Ephrin-A1 seems to be overexpressed in CC tissue samples and associated with poorer OS [72,73,77].

High ephrin-B2 expression correlated with adverse clinical disease stage, larger tumor size, LN metastasis, high MVD, and poor survival in CC tissue samples [75,76].

Table 7 summarizes the role of the EPH/ephrin system in CC tissue samples.

### 4.3. The EPH/Ephrin System as a Treatment Target in GC

As studies continue to prove the tumorigenic properties of various EPH/ephrin system members in GC, several researchers have focused on the development of novel therapeutic strategies targeting their carcinogenic actions over the past decades. In terms of OC treatment, restoration of miR-520d-3p decreased EPHA2 protein levels, thus suppressing tumor growth and migration both in vitro and in vivo, while dual inhibition of EPHA2 in vivo using 1,2-dioleoylsn-glycero-3-phosphatidylcholine (DOPC) nanoliposomes loaded with miR-520d-3p and EPHA2-siRNA had synergistic antitumor effects and greater therapeutic efficacy than either monotherapy alone [78]. The combination of EPHA2-targeting siRNA-DOPC with paclitaxel, on the other hand, resulted in a drastic reduction of tumor growth in vivo in comparison with treatment with paclitaxel and a nonsilencing siRNA [79], while platinum triggered an oncogenic EPHA2-S897 phosphorylation in vivo associated with platinum resistance [80]. Novel EPHB4-based therapeutic approaches against OC include the bidirectional ephrin agonist peptide (BIDEN-AP), which was found to inhibit endothelial migration and tube formation and to effectively suppress invasion and epithelial–mesenchymal transition in OC cell lines. In vivo, BIDEN-AP and its nanoconjugate CCPM-BIDEN-AP significantly hindered the growth of orthotopic ovarian tumors and compromised angiogenesis [81]. JI-101, an oral multikinase inhibitor targeting EPHB4, was tested in a clinical trial to assess its efficacy in OC. Unfortunately, no patient demonstrated a response according to predetermined criteria [82]. PF-06647263, an anti-ephrin-A4 antibody–drug conjugate, achieved sustained tumor regression in OC patient-derived xenografts in vivo [83]. In a phase I study, response to treatment with PF-06647263 did not, however, seem to correlate with ephrin-A4 expression levels [84].

In vivo therapy experiments in mouse orthotopic models with monoclonal antibodies targeting EPHA2 resulted in significant MVD reduction, decreased proliferation, increased apoptosis, and a lower incidence of distant metastasis in EC [58,85].

Table 8 presents the different therapeutic agents targeting the EPH/ephrin system in GC.

## 5. Conclusions

It is established that members of the EPH/ephrin system are implicated in a multitude of molecular procedures, exerting their role in different stages of GC. Consequently, up- or downregulation of each individual EPH/ephrin in gynecological tract tumors influences numerous clinicopathological parameters such as tumor grade, invasion depth, tumor cells’ proliferation rate (assessed by Ki67 index), FIGO stage, hormone receptor status, patient survival, and response to chemotherapy. Therefore, the aforementioned family of biomolecules emerges as potent biomarkers, useful in the fields of diagnosis, prognosis, and disease monitoring in GC. All investigated ephrins exhibited upregulation in OC, EC, and CC, whereas EPHs showed either significant overexpression (OC, EC, CC) or extensive loss (OC) depending on the particular receptor. However, discrepancies between the results reported by different research groups do exist, with studies sometimes reaching contradictory conclusions on the role of some EPHs/ephrins in certain types of GC. EPHA1 expression, for instance, has been reported to be upregulated in OC by several authors [22,34,35,36], whereas Jin et al., concluded that EPHA1 expression is decreased in OC [23]. Of note, Jukonen et al., used seven independent OC datasets, as well as the Cancer Genome Atlas (TCGA), for result validation, and reported variable gene expression for different EPHs/ephrins in high-grade OC [33]. In recent years, large cancer genome studies, such as the TCGA, have searched exhaustively for genes, thus offering invaluable data in terms of results validation. Further research is, therefore, necessary in order to shed light on the impact of certain EPHs/ephrins on carcinogenesis, while researchers may make use of the TCGA and similar datasets to systematically investigate EPHs/ephrins in cancer entities. Given their tumor-promoting or tumor-suppressive properties, EPHs/ephrins could be targeted by therapeutic agents, blocking those members enhancing carcinogenesis or inducing the expression of those suppressing it. Several researchers have already investigated the use of agents targeting members of the EPH/ephrin system, alone or in combination with traditional chemotherapeutic agents, via both in vivo and in clinical studies [58,69,78,79,81,82,83,84,85] and found promising antitumor effects for GC. Anti-ephrin-A4 calicheamicin conjugates, for example, effectively target OC-initiating cells to result in sustained tumor regression [83]; the preclinical data for EC treatment using the antibody drug conjugate MEDI-547 show substantial antitumor activity [85], while the level of EPHA2 expression positively correlates with the therapeutic resistance of CC to epirubicin [69]. Nevertheless, further clinical trials with larger numbers of patients are needed to verify the clinical utility and safety of the agents targeting members of the EPH/ephrin system in the treatment of women with GC, to investigate possible adverse effects following their administration to patients, as well as to assure their effectiveness depending on EPH/ephrin expression by GC cells. A limitation of this review is the nonsystematic methodology in terms of study selection. Even though systematic literature reviews offer the most accurate strategy to detect relevant research works, following rigorous rules and standards, this approach requires a narrow research question that does not cover broader topics, such as the role of the EPH/ephrin system in GC. Another limitation is the possible evidence selection bias, arising from publication bias, given that data from statistically significant studies are more likely to be published. Despite these limitations, the current work represents an extensive and concise review of the literature and includes all the studies conducted on the role of the EPH/ephrin system in GC pathogenesis.

## Figures and Tables

**Table 2:** The role of the EPH/ephrin system in OC cell lines and human xenografts.

EPHs/Ephrins	Cell Lines	Methods	Main Results	Refs.
EPHAs				
**EPHA1**	SKOV3, COV504	RT-PCR, Western blot, cell cycle analysis, cell matrix adhesion/wound healing/invasion/ migration/motility assays	Knockdown suppresses cell cycle arrest, cell adhesion migration, proliferation, and invasion.	[22]
	HO8910, A2780	Cell viability assay, flow cytometry	Low expression levels were reported only in A2780 OC cells. Transfection with EPHA1 plasmid resulted in a significant reduction in the proliferation rate of OC cells.	[23]
**EPHA2**	OVK-18	Immunoblotting, ELISA, immunoprecipitation	Growth promotion.	[24]
	HIO-180, EG, 222, SKOV3, A2780-PAR	Western blot, immunoprecipitation	Overexpression in EG, 222, and SKOV3 OC cell lines. Low to absent expression in A2780-PAR and HIO-180.	[25]
	OVCAR3, SKOV3	Semiquantitative RT-PCR, Western blot	Strong EPHA2 and ephrin-A1 mRNA expression.	[26]
	A2780	Western blot, immunoprecipitation, cell viability/attachment assay, murine tumor xenograft model	Increased expression resulted in the reduction of cell–cell contact, promotion of cell–extracellular matrix attachment, and an increase in anchorage-independent cell growth. Overexpression promoted tumorigenesis, angiogenesis, and metastasis in OC xenografts.	[27]
**EPHBs**				
**EPHB2**	ES-2, OVCAR-3, OV-90, SKOV-3	Semiquantitative RT-PCR, Northern blot	Similarities in RNA expression across OC cell lines and clinical samples. No association between promoter hypermethylation of EPHB2, EPHB3, EPHB4, and OC.	[28]
	A2780wtTP53, A2780mTP53	Affymetrix U133A array analysis	Upregulation in wild-type TP53 OC cell lines.	[29]
**EPHB4**	A2780wtTP53, A2780mTP53	Affymetrix U133A array analysis	Upregulation in the larger pool of mutant TP53 lines.	[29]
	ML5, ML10, MCV 50, HOC-7, OVCAR-3	Western blot, cell cycle analysis, wound healing/ migration/viability/ apoptosis assays, murine tumor xenograft model	Upregulation in OC cell lines correlated with apoptosis inhibition, tumor cell migration and invasion. Progesterone treatment resulted in a dose-dependent reduction in EPHB4 expression, thus promoting apoptosis via activation of the death receptor caspase pathway. Knockdown induced apoptosis and reduced vascularization in murine OC xenografts.	[30]
	A2780, SKOV3	RT-PCR, Western blot, MTT/apoptosis/migration/invasion assays	Downregulation of EPHB4 led to cell growth inhibition, apoptosis induction, and reduced invasive ability in OC cells.	[31]
**Ephrins**				
**ephrin-A1**	OVCAR-3	real-time RT-qPCR, Western blot	NFκB induced ephrin-A1 expression after stimulation with TNF-α and IL-1β.	[32]
**ephrin-A3**	A2780wtTP53, A2780mTP53	Affymetrix U133A array analysis	Upregulation in hypoxia treated A2780mTP53 cells.	[29]
**ephrin-A5**	OVCAR3, OVCAR4, OVCAR8	Treatment with dimeric and monomeric recombinant ephrins	Endogenous ephrin-A5 inefficiently activates EPHA2–pY588 signaling and receptor internalization.	[33]
**ephrin-B2**	A2780wtTP53, A2780mTP53	Affymetrix U133A array analysis	Upregulation in the A2780mTP53 cells.	[29]

**Table 3:** The role of the EPH/ephrin system in OC tissue samples.

EPHs/Ephrins	Tissue Samples	Methods	Main Results	Refs.
EPHAs				
**EPHA1**	8 OC samples, 8 benign ovarian samples	IHC	Upregulation in OC	[34]
	24 OC samples, 4 benign ovarian samples	real-time RT-qPCR	Greater than 10-fold overexpression in OC	[35]
	Ascites of 28 patients with high-grade serous OC, 1 patient with serous borderline tumor	RT-qPCR, survival-associated gene expression analysis	Adverse clinical association	[36]
**EPHA2**	31 OC stroma tissue samples, 8 normal ovarian stroma samples	Microarray data analysis	Upregulation in the stroma of OC	[24]
	5 benign ovarian masses, 10 ovarian tumors of low malignant potential, 79 invasive OC samples	IHC	Overexpression in OC relates to higher tumor grade, advanced stage of disease, and significantly shorter median survival.	[25]
	118 advanced epithelial OC samples	Semiquantitative RT-PCR, IHC	Higher levels of protein expression correlated with a shorter disease-specific survival in OC.	[26]
	24 OC samples, 4 benign ovarian samples	real-time RT-qPCR	Overexpression in OC	[35]
	77 invasive epithelial OC samples	IHC	Overexpression in OC is associated with increased MVD, invasion, high-grade histology, advanced FIGO stage and overexpression of stromal and epithelial MMP-9, epithelial MMP-2, and epithelial MT1-MMP.	[37]
	107 OC samples, 54 ovarian borderline tumors, 45 adenomas	IHC, Western blot, in situ proximity ligation assay	C-EPHA2 was expressed diffusely throughout the tumor in most OC. In OCs, fewer signals of MT1-MMP and N-EPHA2 were observed compared with MT1-MMP and C- EPHA2. No significant difference between MT1-MMP and C/N-EPHA2 interaction was detected in adenomas.	[38]
**EPHA4**	Ascites of 28 patients with high-grade serous OC, 1 patient with serous borderline tumor	RT-qPCR, survival-associated gene expression analysis	Adverse clinical association	[36]
**EPHA5**	61 OC samples, 24 benign ovarian serous tumors, 42 serous borderline tumors, 20 normal fallopian tube samples	IHC	Loss of expression was associated with tumor grade, FIGO stage, and poor outcome.	[39]
**EPHA8**	18 normal ovarian tissue samples, 20 normal fallopian tube tissue samples, 30 benign ovarian tumors, 30 borderline ovarian tumors, 125 OC samples	RT-qPCR, TMA-IHC	High protein level was associated with older age at diagnosis, higher FIGO stage, positive LNs, presence of metastasis, positive ascitic fluid, and higher serum CA-125 level.	[40]
**EPHBs**				
**EPHB1**	74 OC samples, 12 normal ovarian epithelial tissue samples	IHC	Loss of expression was associated with higher tumor grade, metastasis, high proliferative index, Ki67 expression, and significantly worse OS.	[41]
**EPHB2**	115 OC samples	RT-PCR, IHC	OC patients older than 60 years of age exhibited higher expression than younger ones. High levels correlated with poorer OS.	[42]
**EPHB3**	19 normal fallopian tube samples, 17 serous borderline tumor samples, 50 OC specimens	IHC	Expression is significantly reduced in OC compared with normal fallopian tubes and borderline tumors, and is negatively associated with histological grade and FIGO stage of OC.	[43]
**EPHB4**	7 normal ovarian specimens, 85 invasive OC samples	IHC, Western Blot	Upregulation in invasive OC	[30]
	Ascites of 28 patients with high-grade serous OC, 1 patient with serous borderline tumor	RT-qPCR, survival-associated gene expression analysis	Adverse clinical association	[36]
	115 OC samples	RT-PCR, IHC	High levels correlated with poorer OS and poorer response to chemotherapy.	[42]
	72 OC samples	Real-time RT-PCR, IHC	Upregulation in OC, increased with clinical stage and correlated with poor survival.	[44]
**EPHB6**	55 OC samples, 24 benign ovarian serous tumors, 37 serous borderline tumors, 20 normal fallopian tube samples	IHC	High expression was observed in 100% of normal fallopian tube samples, 100% of benign epithelial ovarian tumors, 78% of ovarian serous borderline tumors, and 18% of OC. The expression was significantly associated with grade, TNM stage, and poorer OS, and inversely associated with Ki-67.	[45]
**Ephrins**				
**ephrin-A1**	24 OC samples, 4 benign ovarian samples	real-time RT-qPCR	Expression correlated with poor survival.	[42]
**ephrin-A5**	High-grade OC samples	IHC, tissue microarrays	Overexpression in the most aggressive high-grade OC and upregulation in the high-grade OC cells upon disease progression. High expression was most strongly associated with poor OS.	[33]
	24 OC samples, 4 benign ovarian samples	real-time RT-qPCR	Expression correlated with poor survival.	[35]
	25 OC specimens, 2 normal ovarian tissue samples, 2 benign ovarian tumors	real-time RT-qPCR	Expression was associated with poorer progression-free survival.	[46]
**ephrin-B**	112 OC samples	IHC, Western blot	High-grade OC showed greatest expression. A correlation was found between ephrin-B expression and MVD. Expression was associated with higher rates of disease recurrence and a decrease in OS.	[47]
**ephrin-B1**	162 OC samples	IHC	Upregulation in OC cell lines.	[48]
**ephrin-B2**	72 OC samples	Real-time RT-PCR, IHC	Upregulation in OC, increase with clinical stage, and correlation with poor survival.	[44]

**Table 4:** The role of the EPH/ephrin system in EC cell lines and human xenografts.

EPHs/Ephrins	Cell Lines	Methods	Main Results	Refs.
EPHAs				
**EPHA2**	AN3CA, ECC-1, Ishikawa, HEC1A, HEC1B	Array CGH	Amplification in two of the EC cell lines	[54]
	Ishikawa	Real-time PCR, flow cytometric cytotoxicity assay	Blocking of expression resulted in significant inhibition of EC killing, mediated by Vδ1 γδ T-cells.	[55]
**EPHBs**				
**EPHB3**	AN3CA, ECC-1, Ishikawa, HEC1A, HEC1B	Array CGH	Amplification in four of five EC cell lines	[54]
**EPHB4**	AN3CA, ECC-1, Ishikawa, HEC1A, HEC1B	Array CGH	Amplification in four of five EC cell lines	[54]
**Ephrins**				
**ephrin-A1**	Ishikawa	RT-PCR, Western blot, cell aggregation assay	Stimulation of cell attachment and inhibition of cell aggregation through the EPHA receptor pathway	[56]
	Ishikawa	RT-PCR, Western blot, permeability assay	Intercellular dissociation induction	[57]

**Table 5:** The role of the EPH/ephrin system in EC tissue samples.

EPHs/Ephrins	Tissue Samples	Methods	Main Results	Refs.
EPHAs				
**EPHA2**	85 EC samples	IHC	Overexpression in 47% of tumors. Significant correlation with angiogenesis induction and poorer disease-specific survival.	[58]
	139 EC samples, 10 benign endometrial samples	IHC	High expression in 48% of EEC samples. Significant correlation with high stage and grade, increased myometrial invasion depth, low hormone receptor levels, high Ki-67 expression, and poorer disease-specific survival.	[59]
**EPHBs**				
**EPHB4**	68 EC samples, 16 normal endometrium tissue samples	Real-time RT-PCR, IHC	Significant association with clinical stages, dedifferentiation, myometrial invasion depth, and patient survival rates.	[60]
	26 normal endometrium specimens, 15 hyperplasias, 102 EC samples	IHC	Protein-expressing glandular epithelial cell proliferation in hyperplasias and ECs. Highly significant positive correlation with postmenopausal stage.	[61]
	12 control endometrial samples, 20 atypical EH tissue samples, 34 EC samples	IHC	Overexpression in atypical hyperplasia and hormone positive EC. Positive correlation with ER expression in EC.	[62]
	20 EC samples, 20 normal endometrial samples	IHC	Significant correlation with histological grade and certain clinical stages of EC.	[63]
	EC samples (number not specified)		Significant association with PCNA-labeling index, clinical stage, histopathological grade, myometrium invasion depth, and clinical outcome.	[64]
**Ephrins**				
**ephrin-B2**	68 EC samples, 16 normal endometrium tissue samples	Real-time RT-PCR, IHC	Significant correlation with clinical stage, histopathological grade, myometrial invasion depth, and survival rates in EC.	[60]
	12 control endometrial samples, 20 atypical EH tissue samples, 34 EC samples	IHC	Overexpression in atypical hyperplasia and hormone-positive EC. Positive correlation with ER expression in EC.	[62]
	20 EC samples, 20 normal endometrial samples	IHC	Significant correlation with myometrium invasion depth.	[63]
	EC samples (number not specified)		Significant association with PCNA labeling index, clinical stage, histopathological grade, myometrium invasion depth, and clinical outcome.	[64]

**Table 6:** The role of the EPH/ephrin system in CC cell lines and human xenografts.

EPHs/Ephrins	Cell Lines	Methods	Main Results	Refs.
EPHAs				
**EPHA2**	SiHa, HeLa, C4-i	real-time qPCR, IHC, Western blot, cell proliferation/wound healing/tumor sphere formation/transwell migration assay, murine tumor xenograft model	Knockdown decreased CC tumorigenicity in vitro and vivo. Overexpression promotes CC tumorigenicity in vitro and in vivo. EPHA2 conferred CC cell chemotherapy resistance by upregulating CDK6 expression. Expression levels were correlated with CDK6 levels.	[69]
**EPHBs**				
**EPHB2**	HeLa, C33A	Real-time PCR, Western blot, immunoprecipitation, tumor xenotransplantation	Upregulation in CC and high expression in metastatic CC cell lines. Stem cell state induction. EPHB2/R-Ras signaling is activated in CC.	[70]

**Table 7:** The role of the EPH/ephrin system in CC tissue samples.

EPHs/Ephrins	Tissue Samples	Methods	Main Results	Refs.
EPHAs				
**EPHA2**	158 CC samples, 103 CIN samples, 58 cervicitis samples	IHC	Knockdown decreased CC tumorigenicity in vitro and vivo. Overexpression promotes CC tumorigenicity in vitro and in vivo. EPHA2 conferred CC cell chemotherapy resistance by upregulating CDK6 expression. Expression levels were correlated with CDK6 levels.	[69]
	217 CC samples	IHC	Upregulation in CC	[72]
	206 CC samples	RT-PCR, IHC	High level was significantly associated with OS.	[73]
**EPHBs**				
**EPHB2**	CC samples	Oncomine Database, Human Protein Atlas	Upregulation in CC	[74]
**EPHB4**	62 CC samples	Real-time RT-PCR, IHC	High expression correlated with adverse clinical disease stage, larger tumor size, LN metastasis, and poor survival.	[75]
	90 CC samples, 15 CINs, 15 normal cervix samples	IHC	Overexpression in CC or CIN compared to in normal cervices. Strong expression was correlated with both clinical stage and tumor diameter. Strong expression correlated with MVD in patients with CC.	[76]
**Ephrins**				
**ephrin-A1**	217 CC samples	IHC	High expression was associated with poor disease-free and disease-specific survival.	[72]
	206 CC samples	RT-PCR, IHC	Moderate to high level was significantly associated with OS.	[73]
	378 CC samples, 45 normal cervical tissue samples	Gene expression data and clinical information of CC patients and health controls from the Cancer Genome Atlas and from three datasets of the Gene Expression Omnibus database	Upregulation in CC	[77]
**ephrin-B2**	62 CC samples	Real-time RT-PCR, IHC	High expression correlated with adverse clinical disease stage, larger tumor size, LN metastasis, and poor survival.	[75]
	90 CC samples, 15 CINs, 15 normal cervix samples	IHC	The expression was higher in CC or CIN than in normal cervices. Strong expression was correlated with tumor diameter. Strong expression correlated with MVD in patients with CC.	[76]

**Table 8:** Therapeutic agents targeting the EPH/ephrin system in GC.

EPHs/Ephrins	Cell Lines	Tissue Samples	Methods	Main Results	Refs.
	EPHAs				
**EPHA2**	HeyA8, SKOV3ip1, ES2	91 OC tissue samples	In vivo treatment with miR520d-3p-DOPC and si-EphA2-1-DOPC	Synergistic therapeutic efficacy of dual inhibition in vivo using DOPC nano-liposomes loaded with miR-520d-3p and EPHA2-siRNA in OC, due to miR-520d-3p targeting EPHB2.	[78]
	HeyA8, SKOV3ip1		In vivo treatment with EphA2-targeting siRNA-DOPC	Tumor growth inhibition after EPHA2-targeting siRNA therapy. Significant tumor growth reduction after combination of EPHA2-targeting siRNA-DOPC with paclitaxel.	[79]
	OVCAR3, OVCAR4, OVCAR8	Abdominal ascites fluid	Pharmacological inhibition or knockdown of RSK1/2	RSK1/2 inhibition or knockdown suppressed oncogenic EPHA2-S897 phosphorylation and EPHA2-GPRC5A coregulation, and promoted canonical tumor-suppressive tyrosine phosphorylation and downregulation of EPHA2. The combination of RSK inhibitors with platinum induced GPRC5A^high^ cell apoptosis.	[80]
		85 EC tissue samples	In vitro/vivo treatment with EA5	EA5 suppressed expression and phosphorylation in vitro. The combination of EA5 and docetaxel resulted in a significant reduction in MVD counts, percent proliferation, and cell death.	[58]
	Ishikawa, Hec-1A, KLE		In vitro/vivo treatment with MEDI-547	In vitro treatment with MEDI-547 decreased viability by inducing apoptosis in EC cells. In vivo treatment with MEDI-547 decreased EC growth and distant metastasis, and induced apoptosis in EC tumor cells.	[85]
	**EPHBs**				
**EPHB4**	HeyA8, A2780Cp20		In vivo treatment with the bi-directional ephrin agonist peptide	BIDEN-AP inhibited invasion, epithelial–mesenchymal transition, endothelial migration, and tube formation in OC cells In vivo treatment with (CCPM-)BIDEN-AP led to significant OC tumor growth inhibition, as well as downregulation of epithelial–mesenchymal transition and angiogenic pathways.	[81]
		4 patients in the pharmacokinetic cohort, 11 patients in the pharmacodynamic cohort	Combined JI-101/everolimus treatment in an OC expansion cohort	No serious adverse events and good toleration of JI-101, alone or in combination with everolimus. Possible drug–drug interaction due to increased exposure of everolimus when combined with JI-101. No response per RECIST criteria.	[82]
	**Ephrins**				
**ephrin-A4**		OC tissue samples	In vivo treatment with anti-EFNA4 calicheamicin conjugates	Anti-ephrin-A4 calicheamicin conjugates resulted in sustained tumor regression.	[83]
		16 OC patients	Treatment with PF-06647263 in patients with OC	PF-06647263 treatment response was not associated with expression levels.	[84]

## Data Availability

Not applicable.

## Supplementary Materials

The following supporting information can be downloaded at: https://www.mdpi.com/article/10.3390/ijms23063249/s1. Reference [86] is cited in the supplementary materials.
