# Supplementary material for: The EPH/Ephrin System in Gynecological Cancers: Focusing on the Roots of Carcinogenesis for Better Patient Management
Source: Int J Mol Sci. 2022 Mar 17;23(6):3249. doi: 10.3390/ijms23063249 (PMC8949008; doi:10.3390/ijms23063249)
Supplement: Supplementary file 1 [file ijms-23-03249-s001.zip › ijms-1628242-supplementary.pdf]

International Journal of
Molecular Sciences

MDPI

Review

The EPH/ephrin system in Gynecological Cancers: Focusing on the roots of carcinogenesis for better patients' management

Iason Psilopatis[1,2], Alexandros Pergaris[1], Kleio Vrettou[1], Gerasimos Tsourouflis[1], Stamatios Theocharis[1*]

Supplementary Table S1: Nature of the cells of each cell line according to the Cellosaurus database [1].

Cell line	GC type
**Ovaries**	
SKOV3/SKOV3ip1	Ovarian serous cystadenocarcinoma
COV504	Ovarian carcinoma
A2780/A2780-PAR/A2780Cp20/A2780wtTP53/ A2780mTP53	Ovarian endometrioid adenocarcinoma
OVK-18	Ovarian endometrioid adenocarcinoma
HIO-180	Ovary; surface epithelium
EG	Ovarian endometrioid adenocarcinoma
222	Ovarian mixed germ cell tumor
ES 2	Ovarian clear cell adenocarcinoma
OVCAR3	High-grade ovarian serous adenocarcinoma
OVCAR4	High-grade ovarian serous adenocarcinoma
OVCAR8	High-grade ovarian serous adenocarcinoma
OV 90	High-grade ovarian serous adenocarcinoma
ML5	Ovarian serous cystadenoma
ML10	Ovarian papillary cystadenoma
MCV 50	Ovarian papillary cystadenoma
HOC-7	Ovarian serous adenocarcinoma
HeyA8	High-grade ovarian serous adenocarcinoma
**Endometrium**	
AN3CA	Endometrial adenocarcinoma
ECC-1	Endometrial adenocarcinoma
Ishikawa	Endometrial adenocarcinoma
HEC1A	Endometrial adenocarcinoma
HEC1B	Endometrial adenocarcinoma
KLE	Endometrial adenocarcinoma
**Cervix**	
HO8910	Human papillomavirus-related endocervical adenocarcinoma
SiHa	Human papillomavirus-related cervical squamous cell carcinoma

HeLa	Human papillomavirus-related endocervical adenocarcinoma
C4-i	Human papillomavirus-related cervical squamous cell carcinoma
C33A	Cervical squamous cell carcinoma

References

1. Expasy-Cellosaurus. Available online: https://web.expasy.org/cellosaurus/ (accessed on 11 March 2022)
